# Correlation between anticholinergic burden and behavioral and psychological symptoms in patients with cognitive impairment and dementia

**DOI:** 10.1002/alz.086393

**Published:** 2025-01-03

**Authors:** Sabrina Pistorio, Gianluca Scotto di Tella, Raffaella Merenda, Leonardo Bencivenga, Michela Zanetti, Giuseppe Rengo, Grazia Daniela Femminella

**Affiliations:** ^1^ University of Trieste, Trieste Italy; ^2^ University of Naples Federico II, Napoli Italy

## Abstract

**Background:**

Drugs with anticholinergic properties are frequently prescribed to patients with cognitive impairment. The cholinergic system plays an important role in the learning process, memory, but also in the regulation of emotions. The aim of this research is to investigate a possible correlation between the use of anticholinergic drugs and the risk of developing more severe behavioral and psychological symptoms (BPSD).

**Method:**

Patients with a diagnosis of subjective cognitive impairment, MCI or dementia were recruited from the Geriatric Unit of the AOU Federico II of Naples. Screening tests for cognitive impairment (MMSE, ACE‐R) and functional status (ADL, IADL) were performed. BPSD were evaluated with the Neuropsychiatric Inventory (NPI). The anticholinergic burden was calculated using the ACB calculator. We compared patients at low risk of anticholinergic adverse effects (ACB<3) versus patients at high risk (ACB≥3). Chi‐square test and Mann‐Whitney test were used to compare the two groups. A multiple linear regression was performed to identify factors associated with a higher NPI score. A p value <0.05 was considered statistically significant.

**Result:**

A total of 173 patients (mean age 74±7, 74 men) were included in the study; 132 patients with ACB<3 (low risk) versus 41 patients with ACB ≥3 (high risk) were compared. No statistically significant differences were found between the two groups in terms of demographics (age, sex) and anamnestic variables (education, marital status, family history of dementia, hypertension, diabetes, smoking, dyslipidemia, atrial fibrillation, coronary heart disease, use of alcohol and polypharmacy). Significantly higher NPI scores were found in patients with ACB≥3 (median scores 48.0 vs 15.5, p = 0.00). Furthermore, patients with ACB≥3 showed lower MMSE (median scores 19.0 vs 24.5, p = 0.00) and more IADLs lost. In the multivariate regression analysis, after adjusting for age, sex, polypharmacy and IADLs lost, only the MMSE scores and the ACB score were independent predictors of the NPI score.

**Conclusion:**

In conclusion, the anticholinergic burden might play a role as a risk factor for developing more severe BPSD in patients with subjective cognitive impairment, MCI and dementia, independently of the degree of cognitive impairment.

